# Quantitative trait loci responsible for sharp eyespot resistance in common wheat CI12633

**DOI:** 10.1038/s41598-017-12197-7

**Published:** 2017-09-18

**Authors:** Xujiang Wu, Kai Cheng, Renhui Zhao, Shujiang Zang, Tongde Bie, Zhengning Jiang, Ronglin Wu, Derong Gao, Boqiao Zhang

**Affiliations:** 1 Institute of Agricultural Science of the Lixiahe District in Jiangsu Province, Yangzhou, 225007 People’s Republic of China; 2 Key Laboratory of Wheat Biology and Genetic Improvement on Low and Middle Yangtze River Valley Wheat Region (Ministry of Agriculture), Yangzhou, 225007 People’s Republic of China

## Abstract

Sharp eyespot is a major fungal disease of wheat caused by *Rhizoctonia cerealis* in cool and humid environments worldwide. In this study, 224 single seed descent derived F_13_, F_14_ and F_15_ recombinant inbred lines (RILs) from the cross between CI12633 (a resistant cultivar) and Yangmai 9 (a susceptible cultivar) were assessed for sharp eyespot resistance (*R.cerealis* isolate R0301) in field and greenhouse conditions in three growing seasons. Different agronomic characteristics were also evaluated in the field with no disease infection. All the lines were genotyped with the Illumina iSelect 90 K SNP wheat chip and 101 SSR markers. Sharp eyespot resistance was significantly negatively correlated with heading date and tiller angle, and significantly positively correlated with the diameter of the basal first internode and second internode. Five QTL with a likelihood of odds ratio score of higher than 3.0 were detected on chromosomes 2BS, 4BS, 5AL and 5BS, respectively. These identified QTL may be used in future wheat breeding programs through marker assisted selection for developing sharp eyespot resistant cultivars.

## Introduction

Wheat sharp eyespot, caused by *R. cerealis*, is a serious fungal disease of wheat worldwide, leading to yield losses and threatening food security^1^. Although fungicides can be effective in reducing yield losses from the disease at the seedling stage, the occurrence of fungicide-resistant isolates of the pathogen has increased the need for host resistance to limit sharp eyespot epidemics^2,3^. Furthermore, the fungicides are not effective after the jointing stage due to the difficulty of spraying to the bottom half of the plants. Breeding resistant cultivars is, therefore, an ideal method of controlling wheat sharp eyespot disease.

While many studies have been carried out over the last decade to identify sharp eyespot resistance genes, no wheat germplasm resources with confirmed immunity or complete resistance to sharp eyespot have been found. Partial resistance was found in some wheat cultivars or Chinese landraces such as ARz, CI12633, Shanhongmai and AQ^4,5^. QTL for sharp eyespot resistance were identified on chromosomes 1A, 1D, 2A, 2B, 2D, 3A, 3B, 3D, 4A, 5D, 6B, 7B and 7D, respectively^4,6–9^. Among these QTL, a QTL from chromosome 7D was consistently detected in different populations with the highest phenotypic variation.

High-density linkage maps are very effective for the detection of QTL for important agronomic traits, fine mapping and identification of candidate genes, map-based cloning, and marker-assisted selection^10–12^. For sharp eyespot resistance, however, only a small number of DNA markers (<600) were used for mapping thus some minor QTL could not be detected. The development of high-density wheat single nucleotide polymorphism (SNP) iSelect array which comprises approximately 90000 gene-associated SNPs^13^ makes it possible for mining useful QTL for sharp eyespot resistance through high-resolution maps.

Agronomic traits, such as plant height, heading date, tiller angle, and the diameter of the internode (the first and second), may aggravate or alleviate disease development. For example, one resistant QTL was found to be significantly associated with plant heading time^4^. The close relation was reported between agronomic traits and other resistance genes. In rice, tiller angle showed a significant effect on plant’s resistance to sheath blight which is similar to wheat sharp eyespot^14^. These factors must, therefore, be considered when studying sharp eyespot resistance in wheat.

CI12633 is an *R. cerealis*-resistant winter wheat line. Our study aimed to clarify the relationship between sharp eyespot resistance and the five independent agronomic factors and to identify sharp eyespot resistance QTL and tightly linked SNP markers for use in future marker-assisted selection programs or positional cloning.

## Results

### Performance of the measured traits in three years

**Figure 1:**
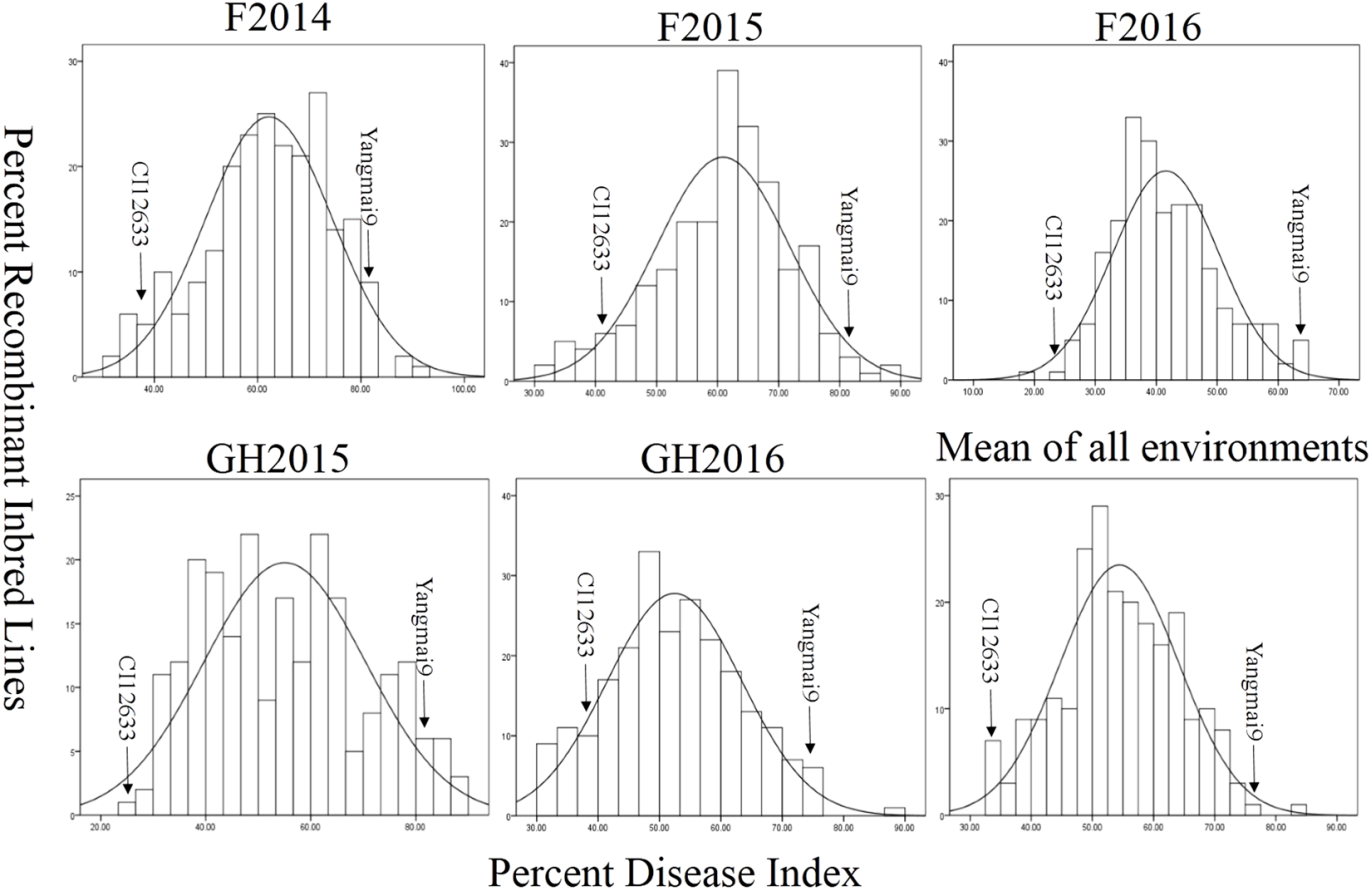
Histograms showing frequency distribution pattern of sharp eyespot disease index (%) values in five environments. F2014, evaluated in field during the 2013–2014 cropping season; F2015, evaluated in field during the 2014–2015 cropping season; F2016, evaluated in field during the 2015–2016 cropping season; GH2015, evaluated in greenhouse during the 2014–2015 cropping season; GH2016, evaluated in greenhouse during the 2015–2016 cropping season; Mean of all environments, averaged data across five environments.

The distribution pattern of the disease index for sharp eyespot in the 224 RILs indicated that resistance to sharp eyespot is controlled by multiple genes and not by a single gene in the CI12633/Yangmai 9 population (Fig. 1). The skewness and kurtosis were all near zero, which indicated that resistance trait in CI12633 exhibited a normal distribution at all the environments. Sharp eyespot symptoms were examined in five environments: field in 2013–2014 (F2014), field in 2014–2015 (F2015), field in 2015–2016 (F2016), greenhouse in 2014–2015 (GH2015), and greenhouse in 2015–2016 (GH2016). CI12633 was rated as resistant with a mean sharp eyespot disease index of 27.79% (11.32–42.77%) while Yangmai 9 was rated as susceptible with a mean score of 83.04% (78.76–86.08%). The disease index for the 224 RILs showed continuous distribution, with ranges of 31.59–92.53%, 30.36–87.65%, 18.35–64.96%, 24.21–88.98%, and 30.02–87.61% in individual environments (Table 1).

**Table 1:** Ranges and mean sharp eyespot disease index for the parents and RILs of the ‘CI12633/Yangmai 9’ population.

Environment	Parents		RIL population			
	CI12633	Yangmai 9	Mean ± standard deviation	Range	Skewess	Kurtsis
F2014	37.42	83.50	62.19 ± 12.32	31.59–92.53	−0.32	−0.35
F2015	25.80	78.76	60.90 ± 10.83	30.36–87.65	−0.37	0.13
F2016	21.66	86.08	41.57 ± 8.69	18.35–64.96	0.52	0.08
GH2015	11.32	81.23	55.04 ± 15.40	24.21–88.98	0.27	−0.9
GH2016	42.77	85.62	52.46 ± 10.97	30.02–87.61	0.18	−0.3

Some disease index detected from RILs which influenced by others pathogen must be abandoned in the data analysis. In the investigation, we found that disease index of sharp eyespot can be infected by wheat powdery mildew and yellow mosaic virus. Usually, disease index of RILs infected by these pathogen was very high.

The analysis of variance (ANOVA) for disease index showed a significant variation for the genotypes and five environments (Table 2). The disease was serious in all environments except F2016 for the small amount of rain in this year which led to air humidity was insufficient for sharp eyespot being hardly occurred even though sprinkled with water every day. Additionally, we found that the correlations for disease index between different trials were all positively significant at the *P* = 0.01 level (Table 3), indicating that the expression of the disease resistance was consistent across the environments thus the data were further used for QTL analysis.

**Table 2:** Analysis of variance of the sharp eyespot disease index of the wheat CI12633 × Yangmai 9 RIL population.

Source	DF	SS	MS	F value	P
Blocks	10	33953.91	3395.391	36.6864	<0.01
Lines	223	320556.9	1437.475	15.5316	<0.01
Experiments	4	181054.8	45263.69	489.0634	<0.01
L × E	892	152580.2	171.054	1.8482	<0.01
error	2230	206390.5	92.5518		
Total	3359	894536.2

**Table 3:** Correlation coefficients among the four trial environments for the sharp eyespot disease index and between disease index and important agronomic traits in the RIL population of the CI12633/Yangmai 9 cross (**Significant at P < 0.01 and *significant at P < 0.05).

	F2014	F2015	F2016	GH2015	GH2016	PH2015	PH2016	HD2015	HD2016	FID2015	FID016	SID2015	SID2016	TA
F2014	1	0.576**	0.370**	0.583**	0.553**	−0.01	−0.04	−0.203**	−0.201**	0.262**	0.221**	0.263**	0.244**	−0.32**
F2015		1	0.507**	0.579**	0.569**	−0.11	−0.146*	−0.155*	−0.158*	0.259**	0.183**	0.298**	0.187**	−0.37**
F2016			1	0.576**	0.590**	−0.10	−0.08	−0.271**	−0.278**	0.482**	0.348**	0.481**	0.341**	−0.06
GH2015				1	0.700**	−0.01	−0.03	−0.204**	−0.205**	0.312**	0.262**	0.337**	0.272**	−0.12
GH2016					1	0.02	−0.01	0.04	0.03	0.366**	0.275**	0.348**	0.287**	−0.15*

### Correlations between disease index with other agronomic traits

The disease index were negatively correlated with heading data (HD) (Table 3). Early heading lines tended to have greater disease index (more susceptible to sharp eyespot) in comparison to lines with late heading dates. The disease index also showed significant positive correlation with first internode diameter (FID) and second internode diameter (SID), indicating that plants with more slender stem are less prone to disease infection. Plant height (PH) is an important trait of wheat, which had no significantly negatively correlated with disease index in each environment (Table 3). A significant negative correlation between disease index and tiller angle (TA) was found in both F2014 and F2015 trials. Lines with greater TA showed less sharp eyespot symptom.

### SNP polymorphism and genetic mapping

The CI12633/Yangmai 9 RIL population was genotyped with the wheat 90 K SNP array which resulted in 2829 polymorphic markers. In addition to the SNP markers, 205 SSR markers were also used to genotype the population. After removing unlinked markers, the resulting map consisted of 2012 markers (1911 SNP markers and 101 SSR markers) spanning 12434.23 cM in length with an average locus density of 6.18 cM/locus.

### QTL mapping analysis in five experiments

**Figure 2:**
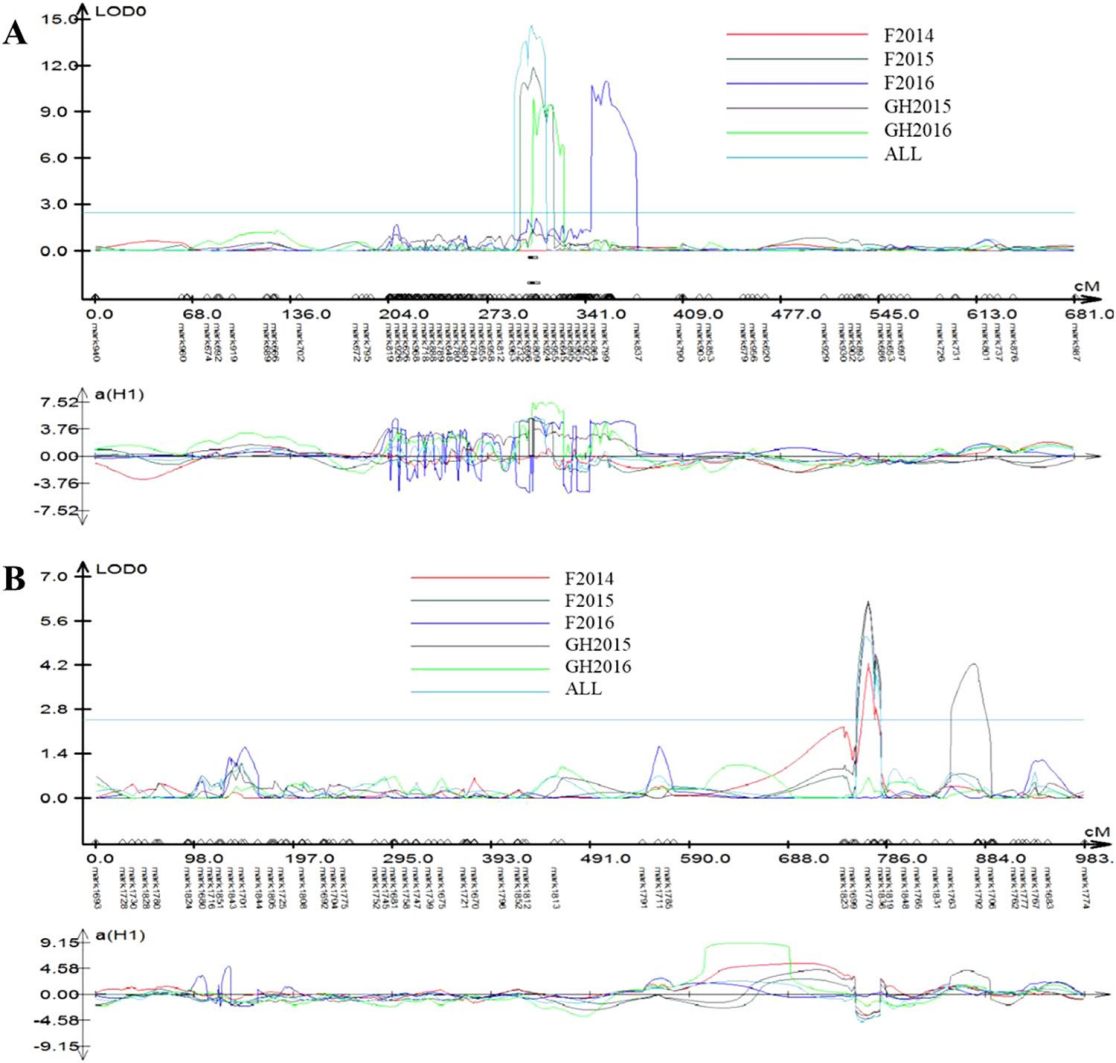
QTL location on chromosome 5AL and 2BS detected using the CIM method across all five trial conditions and the overall mean in the CI12633/Yangmai 9 RIL population. (**A**): 5AL; (**B**): 2BS.

A total of five QTL were identified for sharp eyespot resistance using composite interval mapping, these QTL were identified on chromosomes 2BS, 4BS, 5AL (2) and 5BS, and were designated *QSe.jaas-2BS*, *QSe.jaas-4BS*, *QSe.jaas-5AL.1*, *QSe.jaas-5AL.2* and *QSe.jaas-5BS* respectively. They explained 5.2–37.7% of the phenotypic variation (Table 4; Fig. 2). All resistance alleles were derived from CI12633 except for *QSe.jaas-2BS* which was from Yangmai 9.

**Table 4:** QTL for sharp eyespot resistance in the CI12633/ Yangmai 9 224 RIL population across five environments.

QTL^a^	Marker interval		Position	F2014^b^			F2015			F2016			GH2015			GH2016			Average		
				LOD^c^	R^2d^	Add^e^	LOD	R^2^	Add	LOD	R^2^	Add	LOD	R^2^	Add	LOD	R^2^	Add	LOD	R^2^	Add
*QSe.jaas-2BS*	*RAC875_c730_234*	*RAC875_c16697_1502*	298.21–355.41				12.0	20.7	5.0	11.0	27.7	4.8				9.9	22.2	7.4	14.6	27.9	5.2
*QSe.jaas-4BS*	*RAC875_c49792_228*	*Kukri_c34353_821*	771.6	11.5	32.2	7.5							12.5	37.7	7.0						
*QSe.jaas-5AL.1*	*GENE-3601_145*	*Ku_c21002_908*	767.2	4.3	8.3	−3.6	6.2	10.6	−3.6				6.2	14.7	−4.3				5.1	13.1	−4.5
*QSe.jaas-5AL.2*	*IAAV3043*	*wsnp_Ex_c55777_58153636*	774.5	3.0	5.8	−3.0	4.4	7.8	−3.1				4.6	11.3	−3.8				3.9	9.1	−3.9
*QSe.jaas-5BS*	*wsnp_Ku_c11721_19085513*	*BS00068710_51*	584.41–695.51	3.1	6.2	−3.2	3.0	5.2	−2.9										3.7	7.1	−2.9

^a^QTL were detected with a LOD threshold of 3.0 for declaring significance based on 2000 permutations at *P* = *0.01*; ^b^F2014, evaluated in field during the 2013–2014 cropping season; F2015, evaluated in field during the 2014–2015 cropping season; F2016, evaluated in field during the 2015–2016 cropping season; GH2015, evaluated in greenhouse during the 2014–2015 cropping season; GH2016, evaluated in greenhouse during the 2015–2016 cropping season; Average, averaged data across five environments; ^c^LOD score; ^d^Percentage of phenotypic variation explained by the QTL; ^e^Additive effect of resistance alleles.

Three QTL, *QSe.jaas-2BS, QSe.jaas-5AL.1* and *QSe.jaas-5AL.2*, were detected in three or more environments and explained 20.7–27.9%, 8.3–14.7% and 5.8–11.3% of the phenotypic variation, respectively (Table 4; Fig. 2). *QSe.jaas-2BS* was flanked by *RAC875_c730_234* and *RAC875_c16697_1502*. *QSe.jaas-5AL.1* was located in the interval *GENE-3601_145~Ku_c21002_908*. *QSe.jaas-5AL.2* was flanked by *IAAV3043* and *wsnp_Ex_c55777_58153636*. The *QSe.jaas-4BS*, and *QSe.jaas-5BS* with larger effects for sharp eyespot resistance were both identified in only two environments and explained 32.2–37.7% and 28.7–36.2% of the phenotypic variance, and located in the interval *RAC875_c49792_228~ Kukri_c34353_821* and *wsnp_Ku_c11721_19085513~ BS00068710_51* respectively.

QTL for HD, PH, TA, FID and SID were also analysed. As shown in Table 5, all the QTL for different traits were located in different positions to those for sharp eyespot resistance except for the QTL controlling FID (*QFid-jaas-2BS*) and SID (*QSid-jaas-2BS*). This indicated that the diameter of the wheat basal internode is an important factor in determining sharp eyespot resistance.

**Table 5:** QTL for five agronomic traits related to sharp eyespot resistance in the CI12633/Yangmai 9 224 RIL population across two environments.

QTL	Marker interval		Position	F2014			F2015			Average		
				LOD	R^2^	Add	LOD	R^2^	Add	LOD	R^2^	Add
*QHd-jaas-1AS.1* ^*a*^	*BS00018250_51*	*wsnp_Ex_c48087_53105842*	36.6				4.1	6.6	−18.0	4.0	6.5	−18.0
*QHd-jaas-1AS.2*	*CAP12_c2858_94*	*wsnp_BE586140A_Ta_2_1*	51.3	5.4	7.3	−20.0	10.9	16.3	−28.0	10.9	16.3	−27.7
*QPh-jaas-1AS* ^*b*^	*CAP12_c2858_94*	*wsnp_BE586140A_Ta_2_1*	51.3	8.7	13.5	−14.4	9.4	14.4	−16.2	10.8	17.3	−16.4
*QFid-jaas-2BS* ^*c*^	*RAC875_c730_234*	*BS00059315_51*	295.21–318.31	3.6	8.1	0.1				2.7	17.2	0.2
*QFid-jaas-3BS*	*Excalibur_c602_523*	*BS00040742_51*	480.51–492.21	3.4	11.1	0.2						
*QSid-jaas-2BS* ^*d*^	*RAC875_c730_234*	*BS00059315_51*	295.21–318.31	3.1	7.3	0.1				3.9	23.4	0.2
*Qta-jaas-1BS* ^*e*^	*Excalibur_c581_1220*	*BS00027006_51*	55.0							3.5	38.5	−21.6
*Qta-jaas-4AL*	*BS00107766_51*	*BS00023151_51*	960.9							2.6	21.5	−22.2
*Qta-jaas-5BS*	*TA003586–0637*	*RAC875_c64190_212*	108.6							2.5	6.5	7.7
*Qta-jaas-6DL*	*Tdurum_contig82605_187*	*Kukri_c80163_135*	546.1							2.9	38.2	−21.6

^a^QTL for heading date; ^b^QTL for plant height; ^c^QTL for the diameter of the first internode; ^d^QTL for the diameter of the second internode; ^e^QTL for tiller angle.

## Discussion

### Phenotyping is a critical step for gene mapping

Limited research has been conducted to date on this disease because of the difficulties associated in accurately phenotyping the plant’s response to sharp eyespot^3^. One of the methods is the use of wheat kernels inoculated with the pathogen and subsequent quantitative categorization using a scale of 0 to 5 based on disease severity^15^. This method is dependent on the placement of the wheat kernel, i.e. the infected kernels should be placed close to but not touching the roots of the plants. The direct contact of plant roots with the inoculated pathogen will allow the pathogen to attack the plant persistently, leading to a higher disease index even in resistant plants, particularly when the environmental conditions are suitable for pathogen growth^16^. In this study, the inoculation was carefully conducted to prevent the direct contact between plant roots and the pathogen and all inoculated plants were dug up for the evaluation of disease index. This is a time-consuming and labor-intensive phenotyping method but the reliability of the results was improved. Two other methods of inoculation, toothpick and seedbed inoculation, are generally used by researchers, with many studies finding that toothpick inoculation was more effective^2,6–8^. In this research, however, the toothpick inoculation method could not be used because the sheath of CI12633 and its descendants was too thin and narrow and would break with the insertion of the toothpick.

### QTL for sharp eyespot resistance

A previous study by Chen *et al*.^4^ identified several QTL for sharp eyespot resistance. These QTL located on chromosomes 1A, 2B, 3B, 4A, 5D, 6B, and 7B, respectively. Among these, the QTL detected on chromosome 2BS was also identified in our study, though their precise location was unclear. BLAST analysis of the GrainGenes and WHEAT URGI websites found that the markers *RAC875_c730_234* and *RAC875_c16697_1502* were similar to the SSR markers Xbarc101–2 and barc200, respectively, suggesting that *QSe.jaas-2BS* and *QSe.cau-2BS* may be the same^5,7^. The QTL on Chromosomes 4BS, 5AL (2) and 5BS identified in the current study were nor reported before. Interestingly, the resistance QTL on 2B (*QSe.jaas-2BS*) with larger effect came from the susceptible wheat Yangmai 9. Some resistance gene have been identified previously in susceptible parents. For instance, a rice sheath blight (SB) resistance QTL allele came from the susceptible cultivar Lemont has been successfully fine mapped by Zuo *et al*.^16^. It is more likely that the wheat Yangmai 9 is not highly susceptible to sharp eyespot disease. In Yangmai 9, the resistance QTL *QSe.jaas-2BS* may play a decisive role.

It’s not uncommon that a lot of disease resistance QTL mapped so far do not behave consistently in difference environments. In rice, a similar phenomenon was observed by Channamallikarjuna *et al*.^17^ and Zuo *et al*.^16^ in maping rice SB resistance QTLs. In this study, none of the QTL could be detected in all environments, most likely due to that the outbreak of this disease is easily influenced by environments. Thus, the identification of QTL for sharp eyespot resistance should be based on multi-experiments/environments.

### The association between sharp eyespot resistance and agronomic traits

The association between QTL for sheath blight resistance and agronomic traits in rice has been reported^18^. Little research is conducted on the association between agronomic traits and wheat sharp eyespot^19^. Wheat sharp eyespot pathogens favor cool, humid and shady environments thus is generally more predominant in the lower part of the plant than the upper part. Small internode diameter is conducive to field ventilation and light crossing, thereby changing the growth condition of *R. cerealis*. Wheat varieties such as Shanhongmai, Xifeng, and Limai 16 with small internode diameter are generally more resistance to sharp eyespot^5^. Our experiment confirmed that disease index showed significant positive correlation with FID and SID and the resistant QTL on 2B (*QSe.jaas-2BS*) was associated with the internode diameter. Although significant correlations were observed between sharp eyespot resistance and HD, FID, SID, or TA, more detailed analysis are required to establish any definitive link between these phenological traits and plant’s response to this disease.

In summary, the markers closely linked to sharp eyespot resistance genes identified in present investigation can be used for marker assisted selection in wheat improvement.

## Materials and Methods

### Plant materials

The 224 F_13–15_ RILs used for QTL mapping were developed from a cross between CI12633 and Yangmai 9. Yangmai 9, a high-yielding wheat cultivar grown in the wheat region along the middle and lower reaches of the Yangtze from 2000 to 2007, is susceptible to sharp eyespot; CI12633 is a taller winter wheat line that exhibits a high level of sharp eyespot resistance^5^.

### Sharp eyespot inoculum preparation

The *R.cerealis* isolate R0301 is the dominant strain in Jiangsu province, China, and was provided by Professors Huaigu Chen and Shibin Cai of the Jiangsu Academy of Agricultural Sciences, China. R0301 was activated on freshly prepared potato-dextrose agar (PDA) just before use. Inoculum was produced on sterilized wheat kernels using a method similar to that of Lipps and Herr^15^ and Chen *et al*.^4^.

### Evaluation of disease severity in the field

The reactions of the 224 RILs and their parents to sharp eyespot were evaluated at Yangzhou in Jiangsu province during the 2013–2014, 2014–2015, and 2015–2016 cropping seasons. As humid and wet weather conditions prevail in Yangzhou during spring this area is very conducive for sharp eyespot outbreak. All research materials were sown on October 20^th^ for each of the three years, in a randomized complete block design with three replicates. CI12633 and Yangmai 9 were included every 50 lines in each replicate. Each plot was 0.8 m long and rows were spaced 0.4 m apart; in each row, approximately 50 seeds were sown. To achieve a plant density of 40 plants per row some seedlings were removed. High plant densities provided favorable conditions for the spread of sharp eyespot. In early March, when the weather became warm and wet, the plants were inoculated by placing R0301-colonized wheat kernels on the soil surface close to the plants, but the kernels should not be touched with the plants. The kernels were then covered with earth and water was sprinkled three times a day for the first month in non-raining days. Overall, the weather was very suitable for sharp eyespot outbreak two of three seasons with consistent rainfall occurring throughout the experimental period until the final disease recording.

In early May, when the susceptible parent Yangmai 9 began to succumb to *R. cerealis* infection, all sample plants except the edge plants were dug up and individually assessed for disease (approximately 200–250 wheat stems of each line). The infection types were categorized qualitatively from 0 to 5: 0, no lesion; 1, the lesion appeared on the sheaths rather than stems; 2, the width of the lesion was <50% of the infected stem perimeter; 3, the width of the lesion is >50 and <75% of the infected stem perimeter; 4, the width of the disease lesion is >75% of the infected stem perimeter; 5, white spike or dead plant. Disease index = ((0 × X_0_ + 1 × X_1_ + 2 × X_2_ + 3 × X_3_ + 4 × X_4_ + 5 × X_5_)/[(X0 + X1 + X2 + X3 + X4 + X5) × 5]) × 100, where X _0_ − X_5_ indicated plants with infection types 0–5, respectively^15^.

### Evaluation of disease severity in a greenhouse

To better facilitate *R. cerealis* infection and development, the 224 RILs and their parents were sown in a greenhouse during the 2014–2015 and 2015–2016 seasons. All materials were sown in early November in a randomized complete block design with three replicates. The planting and management patterns were similar to those in the field. A water sprinkler system was constructed over the plants that was used to keep the experimental environment moist. In mid-February, when wheat plants were at the tillering growth stage, R0301-colonized wheat kernels were added to the soil surface and covered with soil. The water sprinkler system and temperature control system were then used to facilitate *R. cerealis* infection and development. When Yangmai 9 began to succumb to sharp eyespot, all the plants were dug up for disease assessment. The disease score was calculated as described above.

### Agronomic trait data

All the RILs and both parents were planted in field conditions in the same randomized block design with three replicates without R0301 inoculation in the 2013–2014 and 2014–2015 seasons. Four traits, heading date (HD), plant height (PH), tiller angle (TA), the first internode diameter (FID), and second internode diameter (SID), were measured for each wheat line. HD was recorded when 50% of the plants in any plot were at heading. PH was measured in centimeters from the soil surface to the tip of the ear excluding the awns at the milk-ripening stage. TA was judged as loose or compact. FID and SID were measured with Vernier calipers once the sheath was removed.

### Molecular marker analyses and map construction

Genomic DNA was extracted from young leaves using a DNeasy Plant DNA extraction kit (QIAGEN). Genotyping was done with the Illumina iSelect 90 K SNP wheat chip containing 81,587 SNP markers. This part work was completed by China Golden Marker (Beijing) Biotech Co.Ltd.(CGMB). Linkage groups were generated with the software Joinmap V4.0 and genetic distances between markers were estimated based on the Kasambi mapping function.

### QTL analyses

QTL analyses were conducted by composite interval mapping (CIM) with the software WinQTLCart 2.5. To declare QTL significant, the threshold logarithm of the odds score was arbitrarily set at 3.0. Adjusted means for percent disease index values each year were calculated before pooling the results from all the years for statistical analyses. The QTL were named according to the International rules of Genetic Nomenclature (http://wheat.pw.usda.gov/ggpages/wgc/98/Intro.htm). If one QTL only detected one time, it will be discarded.

### Statistical analysis

Analysis of variance (ANOVA) analyses within the five seasons of field and greenhouse sharp eyespot data were conducted using PROC ANOVA in SAS 9.1.2 (SAS Institute Inc., Cary, NC, USA). Correlations between experiments were calculated using Excel.
